# Current and Future Trends in Driver Behaviour and Traffic Safety Scholarship: An African Research Agenda

**DOI:** 10.3390/ijerph20054290

**Published:** 2023-02-28

**Authors:** Rose Luke

**Affiliations:** Department of Transport and Supply Chain Management, University of Johannesburg, Cnr Kingsway and University Roads, Auckland Park, Johannesburg 2092, South Africa; rluke@uj.ac.za; Tel.: +27-115594951

**Keywords:** driver behaviour, road safety, Africa, bibliometric analysis

## Abstract

Road traffic accidents are strongly associated with driver behaviour. Africa, as a region, has the highest road accident fatality rate, but there is very little research dealing with this critical issue on the continent. This paper, therefore, sought to establish the state of driver behaviour and road safety scholarship in Africa to determine current research trends as well as potential future research directions. To this end, two bibliometric analyses were conducted, one which considered the issue from an African perspective and the other which considered the broader body of work. The analysis revealed a critical shortage of research related to driver behaviour in Africa. The existing body of research primarily focused on the identification of issues and tended to focus on narrow research problems within limited geographical areas. A need was identified for the collection of broader macro-level data and statistical analyses thereof to indicate regional traffic crash patterns; causes and effects; country-level studies, particularly countries with high traffic fatality rates and low levels of research; cross-country comparisons; and modelling. Future research directions should also include the link between driver behaviour, traffic safety and the sustainable developments goals, as well as policy-related research to determine current and potential future country-level policies.

## 1. Introduction

Traffic crashes are estimated to result in approximately 1.35 million fatalities per annum globally and a further 20 to 50 million people incur non-fatal injuries, many being permanent disabilities [1]. The majority of these crashes take place in lower- to middle-income countries (LMICs), estimated at 93% of the world’s fatalities, but only have 60% of the world’s vehicle population [1]. Chen, Kuhn, Prettner and Bloom [2] estimate that this will cost the world approximately USD 1.8 trillion (in 2010 USD) between 2015 and 2030, with LMICs experiencing economic losses from fatal and non-fatal injuries of approximately USD 834 billion [3]. Most countries’ road accidents cost more than 3% of the Gross Domestic Product [1], although this is estimated at anything between 2% and 8% [4]. Road traffic death rates are the highest in Africa compared to any other world region [1], with an estimated 27 deaths per 100,000 population, compared to only 7 in Europe [5]. Most of Africa is considered to have a high or medium-high death rate from road traffic accidents, with the exception of Mauritius (10.85 deaths per 100,000), Seychelles (10.94), Egypt (11.77) and Tunisia (16.02), which are classified as medium-low [6]. A further 12 countries are classified as having a medium-high death rate, whilst the rest have a high death rate. The highest in Africa is Zimbabwe (also the second highest in the world after the Dominican Republic), with a death rate from traffic accidents of 63.47 per 100,000.

The WHO [1] identifies risk factors in traffic accidents as human-related, infrastructure-related, vehicle-related and inadequate law enforcement. Road Safety Education Victoria [7] identifies the three key areas as human factors, vehicle factors and environmental factors. The government of Jharkhand [8] identifies the causes as human (pedestrian, driver, passenger), vehicles, road conditions and weather conditions. Of these, human errors have consistently been identified as the leading cause of accidents. In the 1980s, Treat [9] found that 70.7% of road accidents were the result of human error, followed by environmental factors and vehicular factors. In associated earlier seminal and often cited work [10], the authors asserted that human errors were a definite or probable cause of 90–93% of the examined incidents, with Green and Senders [11] citing this as “human error [being] the sole cause in 57% of all accidents and was a contributing factor in over 90%”. Other studies found similar results. Salmon, Regan and Johnston [12] cite Sabey and Taylor [13] in finding that driver error, pedestrian error or impairment is the main contributory factor in 95% of accidents. The United Kingdom’s annual road collision statistics suggest that the majority of causes are human-related [14], whilst the National Highway Traffic Safety Administration [15] attributes 94% of causes of crashes directly to the driver. In South Africa, the Road Traffic Management Corporation [16] identifies contributory factors to fatal crashes as human factors (85%), road and environmental factors (9%) and vehicle factors (7%). In Cameroon, [17] found that less than 15% of traffic accidents can be attributed to bad roads, car defects and natural disasters, while the rest were attributable to human factors.

The link between human error and traffic safety has therefore been well established in the literature; however, as identified by the aforementioned authors, human errors can include pedestrians, drivers and passengers. The NHTSA [15] directly attributes 94% of crashes to the driver. The RTMC [16] in South Africa recognises that many of the human errors may be related to pedestrians or other road users (for example, cyclists); however, they indicate that the majority of fatalities due to human error are associated with the driver. Driver behaviour has thus become an important area of research, given the high incidence of driver fault in road traffic accidents. It is, however, difficult to identify the key driver behaviours that contribute most significantly to traffic accidents. The WHO [1] identifies human-related factors as speed; alcohol and drugs; non-use of safety equipment such as helmets, child restraints and seatbelts; and distraction. The WHO also asserts that, because of these factors, age and gender may also be factors, with younger people and males being at higher risk. The report also identifies inadequate law enforcement as a key reason for accidents, arguably used to modify driver behaviour. This is generally acknowledged by African governments, as reflected by enacted laws governing alcohol consumption, use of restraints and speed restrictions, albeit that they vary significantly from country to country across the continent; for example, speed limits in urban areas in South Africa and Botswana are 60 km/h, whereas they are 50 km/h in Kenya and Zambia. Tsala et al. [17] identified human factors as drowsiness, excessive speed, imprudence, inattention, non-respect of road signs and poor overtaking. The RTMC [16], in their South African road fatality statistics, identified the major driving errors as speeding, overtaking in the face of oncoming traffic, intoxication, turning in front of oncoming traffic, disregarding traffic lights and signs, crossing the barrier line, U-turns, following too closely, falling asleep and cell phone use. Road Safety Education Victoria [7] expanded driver behaviour to include deliberate risk taking and distraction to include not just cell phones but also music and friends. The Automobile Association [18] summarised driver behaviour as speeding, distraction, drowsiness, alcohol or drugs, and adds awareness of other users and reckless driving. Moran, Baron-Epel and Assi [19] add defiance of state authorities and low socio-economic status. Mosedale, Purdy and Clarkson [20] add incidents involving pedestrians which are not the pedestrians’ fault and lack of anticipation or awareness (for example, looking but not seeing). Ikram and Mahajan [21] and Haghdoost, Baneshi and Zare [22] add education as a factor influencing driver behaviour, whilst the Transport Department Government of Jharkhand [8] identified the failure to understand road signs. Sabey and Taylor [13] also included inexperience, lack of judgement, aggression, recklessness, frustration and other impairments such as illness and emotional distress. Whilst human behaviours such as speeding, intoxication, attitudes, etc., are generally acknowledged as the key causes of traffic crashes, it is also recognised that the environment within which the driver functions has a strong impact on driver behaviour, for example, the road design may be such that a driver’s behaviour is influenced to reduce speed or drive in a way that is less likely to result in collision [23], or stricter law enforcement may reduce risky driver behaviours [24].

It is thus evident that there are significant driving habits or behaviours as well as factors that influence driving behaviour that influence traffic safety. Despite the extremely high rate of road fatalities in Africa, there is very little research on the topic within this context. The Road Traffic Management Corporation [16] provides statistics in South Africa on the causes of road fatalities. These are generally grouped as human factors, road and environmental factors and vehicle factors. Within each factor, such as human factors, the causes are further identified as jaywalking pedestrians, speed too high, hit and run, disregarding traffic lights, intoxication, etc. The National Transport and Safety Authority [25] collects statistics of traffic accidents in Kenya and also describes the crash as well as the causes of crashes. Statistics are produced (albeit not always readily available) for countries such as Egypt, Ghana, Nigeria and Namibia, although it is not clear whether these statistics identify accident causes, or more specifically driver behavioural issues. Whilst there are thus a few countries that identify behavioural causes of crashes, reports and statistics on behavioural causes of crashes are hard to obtain, and the majority of African countries do not have any publicly accessible statistics on the human (or other) factors underlying traffic crashes. There are several research studies that analyse causes of accidents in African countries [17,26,27,28] and several broader (and older) texts analysing road accidents in developing countries [29], but there appears to be little research that identifies the human, specifically driver, causations of accidents in Africa, therefore making it difficult to identify interventions for what is now regarded as a “silent epidemic on wheels” [30]. Therefore, there is a need to consider the current state of research on driver behaviour and its influence on traffic safety, to identify the current research levels and potential future research directions for this critical topic in Africa. The findings of this paper are therefore expected to provide information to practitioners and policymakers by answering the following questions: (1) what is the level and extent of driver behaviour research in relation to traffic safety in Africa? (2) what are the current research trends? and (3) what are the recommended future research directions in driver behaviour and traffic safety in Africa?

## 2. Materials and Methods

This study aimed at identifying and establishing the current research regarding the interaction between driver behaviour and traffic safety in Africa. An initial scoping review was used to map the literature on the level of traffic accidents or crashes, the causes of traffic accidents and the interaction between driver behaviour and traffic safety in Africa. The scoping literature review was used to identify key concepts, assess the potential nature and extent of the available literature [31] and determine the possible gaps in the literature, for the purpose of guiding both practitioners and policymakers on the critical elements related to driver behaviour and road safety (Pham, et al., 2014). The scoping review provided rigour in identifying the elements related to driver behaviour and traffic safety in general, given that there is limited prior research into the phenomenon in Africa.

Thereafter, a bibliometric analysis was used to determine the research trends in driver behaviour and traffic safety in Africa. Whilst a meta-analysis was considered, the broad range and diversity of topics covered within the African research collection makes the collation of data and findings problematic, making bibliometric analysis appropriate to describe the research trends in the area. Bibliometric analysis is a popular and rigorous technique that enables the exploration and analysis of large amounts of scientific data to determine the “evolutionary nuances of a specific field, while shedding light on the emerging areas in that field” [32]. It is a science mapping technique that presents data on researchers on the topic, their affiliated institutions, journals, countries, collaboration networks and themes using statistics and visualisation techniques. Studies that have used a similar technique include works on road safety injuries [33], road safety education [34] and accident analysis and prevention [35]. In this paper, the bibliometric analysis is presented, firstly, as a performance analysis, which is descriptive, and secondly as scientific mapping, which largely visualises various relationships [32]. The Web of Science database was searched for the terms driver behaviour AND traffic safety AND Africa, and sought journal articles, review papers, book chapters and conference papers. Due to very limited search results (32 initial results), the search was expanded to include driving behaviour OR driving/ driver habit (s) AND road safety. Although the search could have yielded higher results using the term “human error”, an initial investigation indicated a high level of results associated with pedestrian behaviour and was therefore excluded. The final search terms thus read (traffic OR road safety) AND (driv*) AND Africa, which yielded 192 results. As there were few results, these were scanned manually for relevance and a total of 96 items were removed due to lack of African application or results not relevant to the topic. A final list of 96 documents was analysed. For international comparison, the same search terms were used, without the addition of Africa. Over 40,000 results were returned. The high level of results suggested that a manual relevance scan was not possible; therefore, to reduce the results to a manageable base, the results were filtered for the Web of Science subject area of “Transportation”, which revealed a total of 8 461 results. Whilst this may have excluded several important sources and can therefore be regarded as a limitation to the study, the database was sufficiently large to yield enough results for meaningful comparison, given the primary focus on African research. All papers in both searches were downloaded as BibTex files. The R language was used to conduct the bibliometric analysis because it was open-source software, and therefore freely available. The Bibliometrix package was used to analyse the research papers, specifically using the Biblioshiny app, which allowed for the calculation and visualisation of the data. The main information regarding the African-related research is shown in Table 1.

The 96 research documents identified through the bibliometric analysis were related to traffic safety and driver behaviour in Africa, revealing documents for the past 26 years. As per Table 1, the average citations per article is 16.22 with a total of 3682 references. By contrast, an analysis of the global literature using the same search parameters revealed over 40,000 records dating back to the early 1980s, suggesting a very low level of scholarship on the topic in Africa, as well as a relatively new level of research interest. 

## 3. Results

### 3.1. Performance Analysis

**Figure 1:**
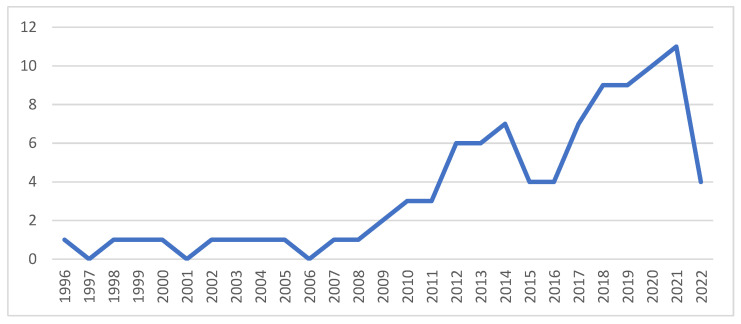
Articles per annum. Source: Author’s own, based on Web of Science search.

Performance analysis was used to determine the contributions of authors, their affiliations, journals which publish the most related articles and countries associated with the contributing authors. Performance analysis accounts for the research constituents [32] and assisted in determining the development of the field of study over time. The results, shown in Figure 1, indicate that between 1996 and 2008, no more than one article was published per year. Since 2008, scholarship in the area has shown an increasing trend, with a high of 11 documents in 2021.

The increasing number of documents in recent years suggests growth in scientific research; however, the level is relatively low, especially considering the enormous body of knowledge elsewhere in the world. The growth can be attributed to the need for research in the area; however, the relatively low numbers of documents also suggest difficulty in attaining data in this area. By contrast, the field has been growing exponentially in the international sphere. From an initial single document in the early 1980s, the number of documents has increased by 16.75% per annum, to a high of 791 documents in 2022. This is more than triple the growth rate of scholarship in Africa. Whilst the field in Africa shows growth, the 2021 output suggests that research targeting the topic of driver behaviour and traffic safety in Africa constitutes approximately 1% of the total global total. Given that the selected sample of 8 461 international papers constitute a small selection of the estimated 40,000 papers, this suggests that the total contribution relating to Africa is considerably lower. 

#### 3.1.1. Publications per Journal

The number of publications per journal reflects the interest in the knowledge field and is reflected in Table 2, using the heading NP (number of publications). The table shows the top 10 journals (12 journals, based on equal results of the lowest four papers). A further two journals, namely Sustainability and the Journal of the South African Institution of Civil Engineering, could also be ranked at the same level, due to each having published two related articles, but were excluded from the final list below, due to lower indices. Table 2 thus indicates the journals that have produced the most articles on driver behaviour and traffic safety in Africa and can therefore be considered to be the most influential journals in this field of study. 

The results reflect that the majority of articles are produced in international journals, with a few prominent South African journals and one Pan African journal. The level of research produced on the continent appears limited. Several of the ten most prominent journals publishing works on the topic in Africa are also most prominent in international publications on the topic, such as Accident Prevention and Analysis, Traffic Injury and Prevention, IATSS Research, Transportation Research Part F-Traffic Psychology and Behaviour and the Journal of Transportation Safety and Security. There are, however, several journals that do not feature prominently in publishing on the research area in Africa, notably Transportation Research Record, the Journal of Safety Research and Transportation Research Part A (Policy and Practice), Part B (Methodological) and Part D (Transport and Environment), suggesting that research on the topic in Africa has not yet considered methodological aspects in depth, nor considered the broader policy aspect required to ensure that driver behaviour aspects are addressed in traffic safety. 

#### 3.1.2. Total Citations and Impact per Journal

The productivity and influence of the published research on driver behaviour and traffic safety in Africa were measured using the h-index of the journal. The h-index measures the productivity and impact of a researcher or group of researchers [36], thereby providing an indication of the influence of work. The top 10 (12, due to equal impact) publications were ranked and are shown in Table 2. The most influential source was Accident Analysis and Prevention, with an h-index of 7 and an impact factor (g-index) of 9, calculated since 1999. This was followed by Traffic Injury Prevention, with an h-index of 6. The International Association of Traffic and Safety Sciences, the Journal of Transport and Health and Transport Research Part F: Traffic Psychology and Behaviour followed, each with an h-index of 3. The high level of influence of Accident Analysis and Prevention since 1999 suggests a dearth of research in the earlier years. The research topic has attracted far more scholarship in the past five years.

The h-indices from the top 25 international journals all exceed those of the top 10 considered in African-related works, suggesting a low level of maturity as well as broader research appeal, perhaps related to the specialist or niche research areas.

#### 3.1.3. Source Growth

**Figure 2:**
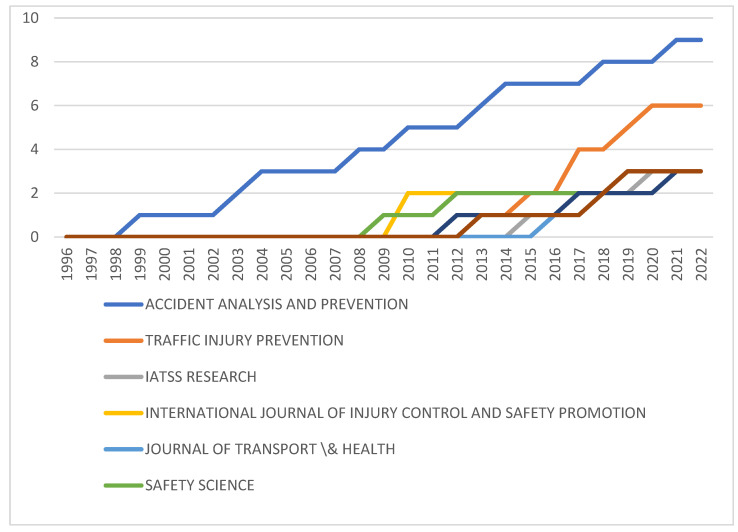
Source Growth. Source: Author’s own, based on Web of Science search.

For the relevant research in Africa, the top five journals since 1996 were examined. Transportation Research Part F: Traffic Psychology and Behaviour provided the earliest publication, followed by Accident Prevention and Analysis in 1998. The source growth of the various journals are shown in Figure 2. A higher level of growth started in 2008, with the journals Accident Prevention and Analysis and Traffic Injury Prevention currently leading the way. Further growth is observed from 2016; however, the research output remains extremely limited. Many of the articles from the journal Accident Prevention and Analysis are related to niche country-level studies of specific aspects, such as the road safety effect of anger and impulsivity of students at a university in South Africa [37], traffic accidents on a specific road segment in Cameroon [38] and superstition amongst taxi drivers in South Africa [39], suggesting issue identification; although some research focused on broader-level comparisons such as works by Nordfjærn, Jørgensen and Rundmo [40] and Factor, Mahalel and Yair [41], providing broader perspectives, these are already somewhat dated. Analysis of more recent research indicates similar patterns, with the focus on niche themes in specific countries, with only one paper considering a systematic review of the economic burden of road traffic injuries in sub-Saharan Africa [42]. The top publications all have high impact factors (for example Accident Analysis and Prevention has a Web of Science impact factor of 4.993 [43]) suggesting broad interest and a wide readership, thus demonstrating the interest in the field.

From the broader international perspective, growth in the research output started in the 1990s for journals such as Accident Analysis and Prevention and the Journal of Safety Research. For the top five journals, exponential growth started in the mid-2000s, with high levels of growth also shown for Traffic Injury Prevention, Transportation Record Part F (Traffic Psychology and Behaviour) and Transportation Research Record. 

#### 3.1.4. Most Influential Authors and Affiliations

A total of 282 authors contributed to the 96 identified articles. Only 14 authors (0.3%) were single authors, suggesting that the majority of the research achieved in this area is the result of collaboration. A ranking of the authors, based on productivity and performance, was conducted to determine the top 25 authors as well as some of the research trends within the area of driver behaviour in Africa. These rankings are shown in Table 3 and are based on total citations as well as the h-index.

Rundmo T was the most influential author, with a total of 262 citations, an h-index of 4 and an impact factor of 3 (g-index), with publications extending from the year 2009 and a relatively consistent output level across the years. Rundmo T also has the highest h-index (4), followed by Bachani AM, Hyder AA, Jones M, Nordjaern T and Renner W, each with an h-index of 3. Most of the top 25 researchers have been publishing for some years, with recently joined authors, indicating that the field is not necessarily attracting new researchers. In the broader research output, there are several authors with high impact factors (h-index above 20), including Abdel-Aty M, Yang H, Watson B, Lajunen T, Li X and Li Y. Over 200 authors joined the research cohort in 2022, suggesting a massive growth in research interest in the field.

#### 3.1.5. Author Affiliations

Author affiliations provide an indication of the institutions and countries where the authors are working. Over 140 institutions are represented. Of the top 30 institutions (with over three articles per institution), shown in Table 4, 9 are South African, 2 are from Nigeria and 2 are from Ghana. Other African countries represented on the top 30 list were Benin, Kenya, Malawi and Zambia. From outside Africa, the most represented countries were, in order, the United States, the United Kingdom, Norway and the Netherlands. 

Top author affiliations in the international research sphere include universities from Australia (Monash, Queensland University of Technology), the Netherlands (Delft University of Technology), China (Southeast, Tongji, Beijing Jiaotong) and the United States (Central Florida, Berkley, Michigan and Texas A&M)

**Figure 3:**
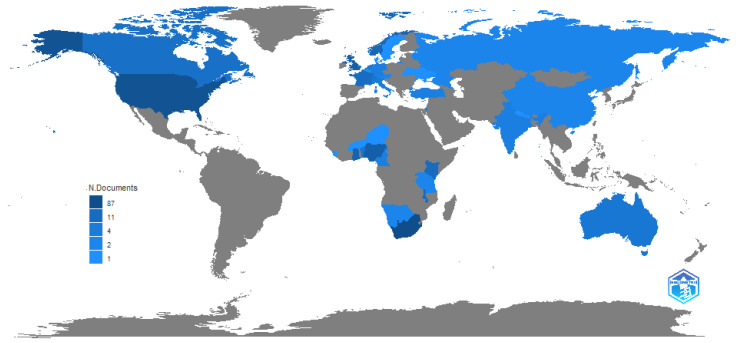
Scientific production per country. Source: Author’s own, based on Web of Science search.

An overview of the scientific production per country indicates that the United States is the biggest producer of articles related to driver behaviour and road safety in Africa. Of interest is that, although Africa participates actively in the research, there are only a few key clusters in southern, eastern and west Africa, with no research arising from central and north Africa. This is illustrated in Figure 3.

The most influential countries were South Africa, the United States, France, Norway and Ghana. Only 4 of the top 10 countries shown in Table 5 were African, again pointing to a dearth of African scholarship in the area. Where research is conducted, these often attract high citation rates, suggesting that researchers are reliant on a small number of articles for local knowledge of the study area. This suggests the need for a far broader range of local-based information.

The most influential countries from the broader international research cohort are, in order, the United States, China, Australia, Canada, the United Kingdom, the Netherlands, Sweden, Germany, France and Spain. There is a strong influence from countries which have relatively good traffic safety records (from North America and Europe), suggesting a level of refinement in the research field. It is also evident from the analysis that there are no African countries featured in the top 35 producing countries; however, Ghana, Egypt, Nigeria and South Africa do feature in the top 50.

### 3.2. Science Mapping

#### 3.2.1. Country Collaboration

**Figure 4:**
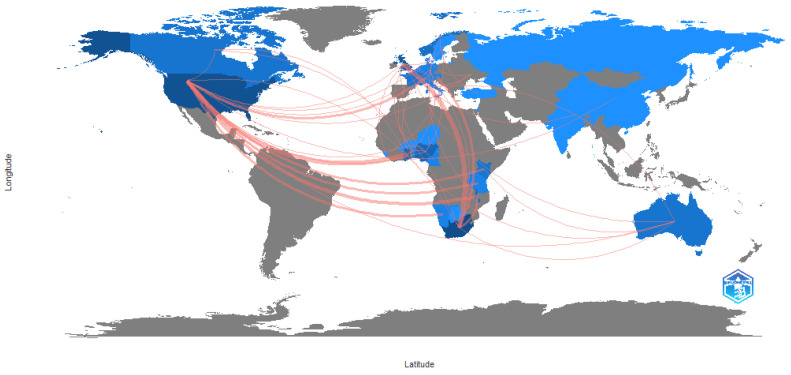
Country collaboration map: African related studies. Source: Author’s own, based on Web of Science search.

To investigate the relationships between researchers, institutions and countries in a particular area of research, science mapping can be used [32]. Science mapping makes use of techniques such as citations, co-citations, thematic clusters, co-word and co-authorship analysis. Collaboration in the area of driver behaviour and traffic safety in Africa mainly took place between Africa and Europe and/or the United States, as is reflected in Figure 4. For the southern and eastern African countries, collaboration was relatively evenly spread between the USA and Europe (mostly the UK), whilst west Africa had fewer collaborations with Europe and stronger ties with the USA. It is also noteworthy that, although present, there is very little intra-African research collaboration.

**Figure 5:**
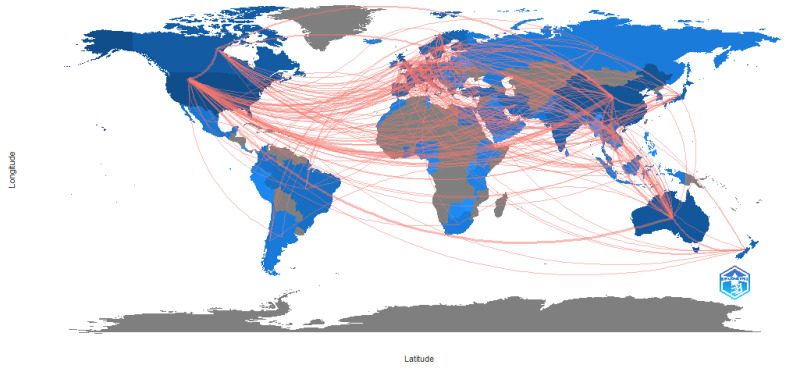
Country collaboration map: Global perspective. Source: Author’s own, based on Web of Science search.

The results also show that there is not a broad level of collaboration in research. Clusters tend to be isolated, with low levels of cooperation between a few selected institutions. By contrast, the volume of research on driver behaviour and traffic safety in the broader sphere is considerable and linkages are reflected in Figure 5, which shows strong linkages between North America, Europe and Australia. The map also shows a general pattern of scholarship emanating from South America and Asia. Collaborations are shown with Africa; however, it is evident that research collaborations with Africa are limited to southern Africa and pockets within east and west Africa. Overall, very little research is produced in Africa.

#### 3.2.2. Co-Citation Analysis

**Figure 6:**
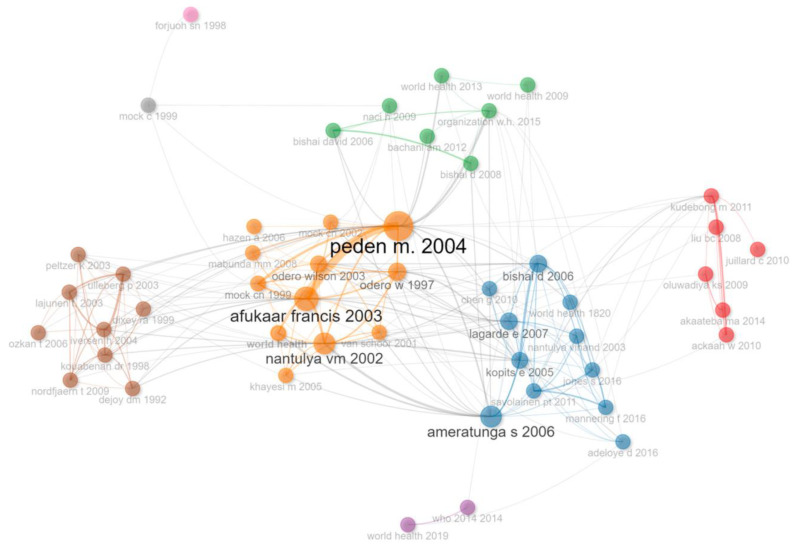
Co-citation analysis. Source: Author’s own, based on Web of Science search.

Co-citation analysis can assist in identifying the most cited articles, thus revealing the most influential and foundational research works [32]. There are three key clusters that can be identified within the extracted documents, as well as two smaller clusters. These are illustrated in Figure 6. One cluster is led by Peden M and relates to factors associated with injury prevention. Another is led primarily by Ameratunga, includes Lagarde E and relates to road traffic crashes, injuries and economic burden. A third cluster has no clear leader but includes works by Dixey R A, Nordfjærn T and Kouabenan D R and relates to cultural influence on driver behaviour. Smaller structures (green and red) relate to strategies to prevent crashes and vulnerable road users whilst stand-alone themes include the WHO, Mock C and Forjuoh S N and primarily relate to injuries and trauma.

From an international perspective, two main clusters and one smaller cluster were formed. The first cluster is led by Reason J and focuses on road safety campaigns and interventions. The second is led by authors such as Milton JC, Savolainen PT and Mannering F and relates to data and statistical analyses and modelling. The much smaller cluster consists of older works by authors such as Richards PI, Lighthill MJ and Gipps PG and focuses on traffic models.

#### 3.2.3. Co-Word Analysis

One of the science mapping techniques that can be utilised is co-word analysis, which uses the co-occurrences between words used within the documents to identify relationships and interactions between words to determine current themes as well as to attempt to predict future research directions [44]. In this analysis, author key words, keywords plus, titles and abstracts were used to identify the most frequently used words. These were tested independently, and it was found that all searches yielded highly similar results and the most used words could therefore be identified based on the collective results. As expected, these 100 most used words tended to relate to roads, traffic, drivers, safety, accidents, fatalities and related terms such as road safety and driver behaviour. Also evident amongst the top 100 words were those related to alcohol/intoxication, driver attitude, pedestrians, vulnerable road users, male/female drivers, culture, drug use, enforcement, child restraint, motorcycle crashes, public transport and auto-rickshaw operators. Whilst the 96 documents could not provide a strong indication of themes other than broad perspectives on road safety and driver behaviour, the identification of issues such as alcohol and drugs suggest key behavioural issues within the theme, as does gender identification and culture. The mentions of pedestrians, motorcycles and auto-rickshaws also help to identify parties other than drivers that may either exhibit behaviours that could result in road safety issues or could be vulnerable due to behaviours towards them. From a methodological perspective, data and data availability are mentioned along with logistics regression, thus not providing significant evidence of methodological approaches used to analyse driver behaviour and road safety in Africa. The low level of methodological method mentions suggests that not enough research has been conducted to discover preferred methods, implying an emerging research area.

The international research shows similar patterns with regards to aspects such as alcohol and drugs, age and novice/older drivers, attitudes, speed, distractions, sensation seeking and pedestrians. Included in these words are several words suggesting technological developments in the field such as driver simulation, signalised intersections and networks, autonomous vehicles and automated driving and machine learning. Eye movements and prediction and situation awareness are also concepts not considered in the African-related literature. Methodologically, there are several entrants to the list such as meta-analysis, theory of planned behaviour, multinomial logit model, data mining, logistics regression, structural equation modelling, naturalistic driving studies and data and data science suggesting broader study fields, wider data ranges and greater maturity in research in the field.

A word trend analysis was also used to assist in determining word growth. The period considered was from the year 2010 to 2022, given that the majority of the growth in the research area was after 2010. The top five words in each year were considered. Several words were found consistently over the period under review, as expected, such as road traffic, safety, injury, crash, etc. There are several words that have, however, only emerged in more recent years such as driver behaviour and risk perception, which emerged in 2014 as trending terms. Terms such as alcohol began to emerge in 2016, as did public health. Links to the sustainable development goals only started to emerge in 2019, and terms such as motorcycle drivers and child restraints only emerged as trends in 2020. These results suggest that many key concepts are still in early-stage research development. 

**Figure 7:**
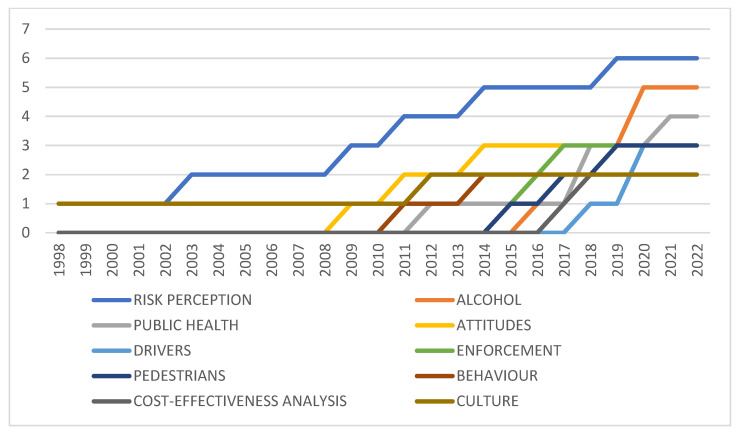
Author keyword frequency. Source: Author’s own, based on Web of Science search.

Globally, these patterns tend to be similar, but emerge earlier. Several other trends that can be observed in the early years of research are terms such as child restraints and safety belts. In the mid-2000s, research relating to cell phone use and driving started to emerge. Themes that remain prominent throughout the 2000s to date are alcohol and drugs, age, dementia and Alzheimer’s and sleep deprivation. Themes that start to trend in more recent years are themes related to technologies such as autonomous vehicles. Trends that show up in both data sets since the turn of the century also include legislation and law enforcement. It is, however, noted that terms related to a word frequency analysis also assist in identifying word growth over time. This identifies some of the themes and key concepts in the research field. Several expected terms were excluded from the results such as developing countries, safety, injuries, accidents and country or region names. Based on author-identified keywords, the results are depicted in Figure 7, and show key research trends such as alcohol, attitudes, enforcement, culture and behaviour as dominant themes, although it is noted that growth trends are relatively low in all themes, based on the general low level of research in the area. Inclusion of excluded words would place injury and road safety as the top two words.

Similarly, the international comparison yields risk as the term with the most significant growth, especially in recent years. These are followed by, in order, behaviour, drivers, model, crashes, performance, accidents, impact, age, speed, time and attitude. The results suggest that there is a higher focus in Africa on alcohol, pedestrians, culture and public health, as expected from priority issues within the continent.

#### 3.2.4. Co-Occurrence Networks

**Figure 8:**
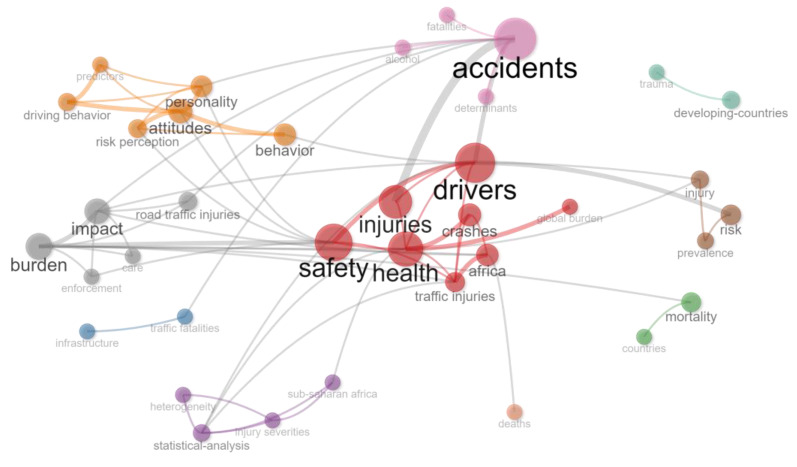
Co-occurrence network: Africa. Source: Author’s own, based on Web of Science search.

**Figure 9:**
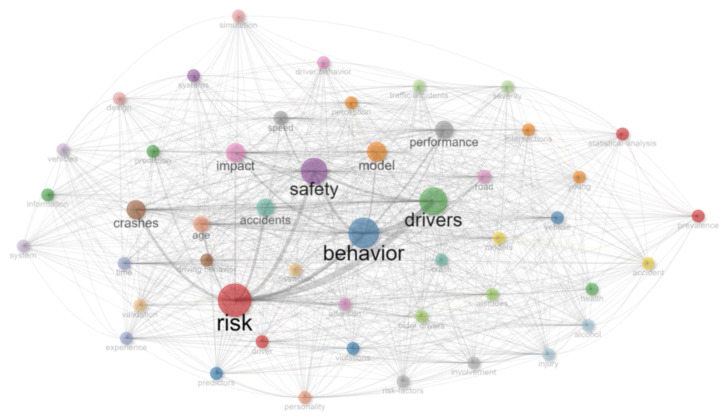
Co-occurrence network: Global. Source: Author’s own, based on Web of Science search.

A co-occurrence network shows clusters of themes, as defined by the authors through their keywords. Author-defined keywords were used over index keywords (which may use subject headings to group synonymous terms) as it is assumed that author-defined keywords are the key description of the work. This enables the visualisation of links between themes and the topics covered in a cluster. The co-occurrence networks for Africa and globally are shown in Figure 8 and Figure 9. The Biblioshiny default settings using the Walktrap Clustering Algorithm were used, with the minimum number of edges set at 2 and the number of nodes at 50.

The African research indicates four key clusters in the co-occurrence network, one related to drivers, injury and safety. A second cluster considers the health burden and the impact of crashes. A third considers accidents, fatalities, alcohol and related aspects in developing countries and a fourth considers attitudes, personality and behaviour. In an initial analysis using the Walktrap Clustering Algorithm, the international literature was clustered around two key themes, one related to drivers, safety and risk and the other related to behaviour and impact. To provide greater resolution, further analysis was conducted using InfoMap, which also indicated very high levels of co-occurrence, with multiple themes emerging, the most prominent of which were risk, behaviour, safety, impact and drivers. There are very strong relationships between the multiple clusters, suggesting both a mature study field and the cross-cutting and interrelated research areas in the knowledge field, whereas the African-related literature tends to have weak linkages between clusters, most likely associated with the low level of scholarship, the relative newness of research and the isolated research topics in the area.

#### 3.2.5. Emerging Themes and Trends

In bibliometric analysis, using the Web of Science database, the Keyword Plus algorithm (considered to be more broadly descriptive than author’s keywords and effective in measuring the knowledge structure of the scientific field [45]) can be analysed statistically to determine the centrality and density of themes. Centrality refers to the degree of interaction that a word cluster has with other parts of the research network. The stronger the link, the greater the significance of the research theme in the development of the field. The stronger the links, the more central the position and the more significant the value of the cluster to the research community [46]. Density refers to the strength of the internal linkages between clusters of words and envisages the ability of the cluster to grow over time (ibid.). This indicates how key thematic areas develop and evolve. Themes can then be classified as:

- Niche themes are developed but have low centrality and are considered to be peripheral or isolated.
- Emerging or declining themes have low centrality and low density, implying a lack of development.
- Basic themes are general and broad; although central, they are less developed than the motor themes.
- Motor themes are considered to be fundamental, core or mainstream themes, and are central and developed [46,47].

**Figure 10:**
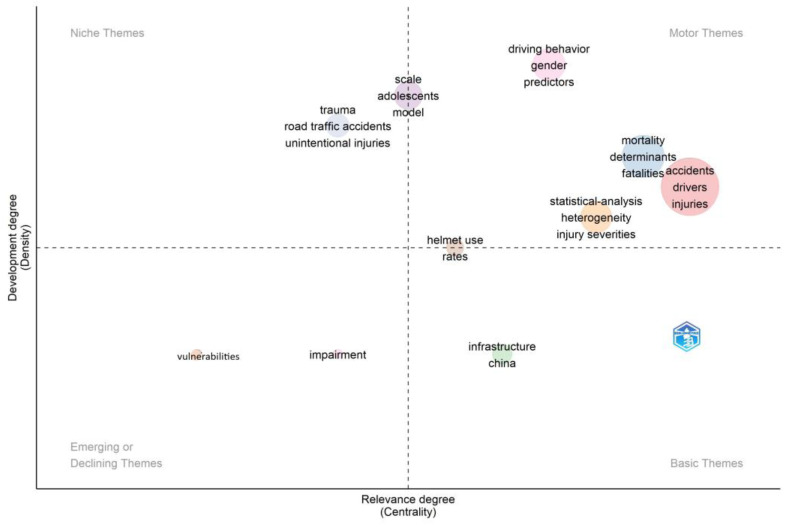
Thematic map of research on driver behaviour and traffic safety in Africa. Source: Author’s own, based on Web of Science search. (Walktrap Clustering Algorithm, Minimum Cluster Frequency 5/1000 documents, 250 keywords.)

**Figure 11:**
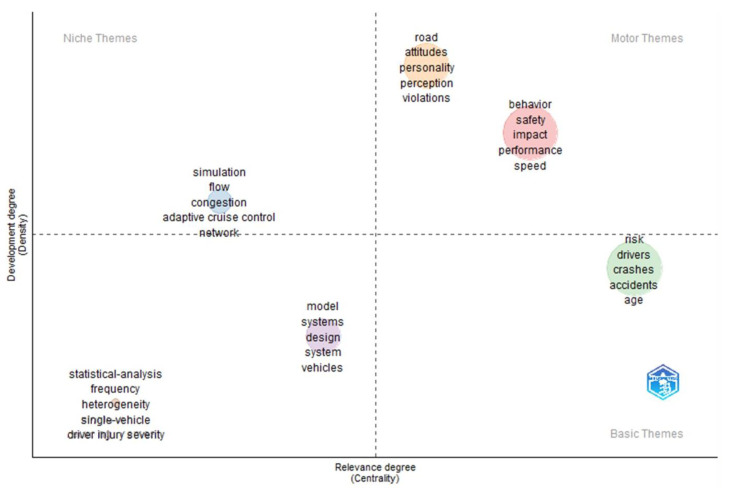
Thematic map of driver behaviour and traffic safety globally. Source: Author’s own, based on Web of Science search (Walktrap Clustering Algorithm, Minimum Cluster Frequency 5/1000 documents, 250 keywords).

These themes are illustrated in Figure 10 (African-related research) and Figure 11 (global research).

Central themes are expected: driving behaviour, gender, determinants of accidents and fatalities, injuries and severity thereof. Infrastructure is considered to be a central, but undeveloped theme, which could relate to the response of drivers to infrastructural issues. Isolated themes that are emerging or declining relate to impairment and vulnerabilities. Some themes that are isolated are adolescents and unintentional injuries.

By contrast, the themes in international research relating to attitudes, personality, behaviour and impact are well developed and central. Age is considered a central theme, although undeveloped. Simulations, flow and networks are isolated developed themes, and model and system designs and statistical analyses are emerging or declining themes.

#### 3.2.6. Top 10 Cited Papers

The top 10 cited papers were analysed based on the local citations per article. These are shown in Table 6. These results reflect that research into the interaction between driver behaviour and traffic safety is extremely limited. In many cases, driver behaviour is only alluded to as part of the results. In addition, the topics tend to be either very broad (e.g., causes of road fatalities) or, by contrast, very narrow (motorcycle riders). In all instances, the field of application is narrow, usually a specific area within a specific country. 

Analysis of the top 10 globally cited articles on the topic in Africa shows somewhat different results, with several papers considering comparisons across countries. These include Lund and Rundmo [57], who examined traffic safety, risk perception, attitudes and behavioural differences between Ghana and Norway. Lagarde [48] broadly compared road safety issues across continents. Nordfjærn et al. [40] considered a comparison of attitudes between Norway, Russia, India, Ghana, Tanzania and Uganda, while Wesson et al. [58] considered traffic safety from a broad low- to middle-income country perspective. Although Factor et al. [41] considered Israeli drivers, they found differences in accident involvement between people of European and American origin, and those from Africa and Asia. In the total list of research on driver behaviour and traffic safety in Africa, driver behaviours were influenced by factors such as superstition, cultural beliefs, risk perceptions, distractions, alcohol and drugs, undiagnosed disorders such as sleep apnoea and ADHD, anger, road rage and impulsivity, and fatalism and personality. The key themes of the African research relate to the burden of the problem, the cultural influences and reasons underlying accidents (reasons for driver behaviour). In general, most articles use qualitative techniques or descriptive statistics to analyse and describe the results. Several studies have considered the cost-effectiveness of road safety interventions using cost-effectiveness analyses (CEAs). A small number of articles have used regression techniques to describe the associations between, for example, demographic attributes, cultural aspects or driver attitudes and traffic safety. These models tend to be limited to a few isolated elements, thus not describing the broader perspective within the country of application or, indeed, the region. 

From an international perspective, the focuses of most of the top 10 locally cited papers show some similarities with the African-focused research. There are several which consider age and experience [59,60,61], enforcement [62], speed and related driving habits [63,64,65], pedestrians [66], naturalistic driving [67] and distractions [68]. Globally cited articles tend to take a broader perspective and focus on aspects such as methodological analyses of statistics [69], analysis of highway data [70,71,72,73] and speed and sensation seeking article reviews [74,75], although there are some more specific topics such as cycling [76], lane changing [77] and parking [78]. 

In general, the most highly locally cited sources tend to be related to specific driving behaviour characteristics, whilst globally cited articles tend to focus on broader aspects, namely country comparisons (African-related studies) and accident statistics or review papers (international studies), suggesting a need for broad-based studies covering a wide range of driving-behaviour-related studies.

## 4. Discussion

Africa is the region in the world which has the highest average accident rate and the highest number of fatalities per 100,000 population [1], indicating a considerable need for intervention. Yet little is known about the issue. Whilst there are some accident statistics in a few countries, in general, data on accidents and traffic are hard to obtain, with Adeloye et al.’s [79] systematic review and meta-analysis of traffic crashes, injuries and deaths identifying studies from only 15 of the 54 countries in Africa. Whilst there are a few studies originating in countries with high road accident fatality rates, e.g., Nigeria, Cameroon, Ghana, Egypt and South Africa, there are broad tracts of Africa with very high accident rates which do not reflect any research (see Figure 3). Where research interest has been recorded, the levels remain critically low. Figure 2 indicates that, although research interest is growing over time, this growth level is very low. Only 96 papers were produced on the topic of driver behaviour in road safety in Africa, compared to a global research interest in excess of 40,000 papers. Whilst the bibliometric analysis did not allow for statistical correlation between research and accident rates, the results from these two bibliometric profiles were contrasted to the accident rates reflected in the World Life Expectancy [6] website and appear to reflect that higher levels of research are associated with road traffic crash and fatality rates. Policy and other interventions require data to determine road safety challenges and opportunities [80] or, as the maxim states, ”if you can’t measure it, you can’t manage it”. As the cause of most road safety accidents is attributed to driver behaviour, it is important to understand the nature of the behaviours in order to manage this. By implication, a lack of research into the types of behaviour suggests that appropriate interventions cannot be identified and therefore implemented. Furthermore, it is important that both broad-level and country-specific research is conducted. The results of this analysis reveal that there are several differences between driver behaviours across cultures and countries [19,40,57], suggesting that interventions need to be based on the driver behaviours exhibited within specific environments. 

An analysis of the journals that publish most frequently on this research topic both in Africa and internationally indicates that there are many articles published in traffic- and accident-related journals, as well as medical journals, although there are some broad-range journals such as the Journal of Transport Geography, the South African Journal of Science and Sustainability. A comparison with the top 10 journals internationally includes journals relating to policy, methodological aspects and environmental aspects. Although the African list includes the journal Sustainability, environmental and sustainability aspects are under-researched in this research cohort, especially considering the sustainable development goals (SDGs) related to road safety. In particular, the United Nations declared the years 2010 to 2020 as the Decade of Action for Road Safety, and two of the SDGs relate directly to road safety. Goal 3.6 is “By 2020 halve the number of global deaths and injuries from road traffic accidents” and goal 11.2 is “By 2030 provide access to safe, affordable, accessible and sustainable transport systems for all, improving road safety, notably by expanding public transport, with special attention to the needs of those in vulnerable situations, women, children, persons with disabilities and older person” [81]. Little research appears to consider road safety from a sustainable development perspective. Moreover, the relative lack of focus on methodological aspects suggest that research is emergent and has considerable scope for development. Although several papers consider interventions to modify selected driver behaviour, the absence of policy journals in the top 10 journal list suggests that the topic has yet to be considered from a policy perspective in Africa. The h-indices of the African-related works on the topic are relatively low, suggesting that the research lacks broad-based appeal, perhaps because of applications that are too specific or too niche, for example, traffic safety on specific roads or within specific regions. Whilst the topic of driver behaviour and traffic safety in Africa is of considerable global interest, as is evidenced by the World Health Organisation, the United Nations, Lagarde’s [48] work, the Sustainable Development Goals, amongst others, research in the area has low citation rates, suggesting that the focus has not started to address the key or critical research gaps. A comparison of the source growth reinforces this by indicating an international focus on prevention and behavioural change, whilst the African-focused research tends to be on identification of issues within particular environments.

Table 1 shows that the majority of African-focused research is co-authored, and analysis of the countries of production (Table 6) as well as the country collaboration network (Figure 4) suggests that there is considerable international input into the research in Africa. However, the relatively low level of African country participation indicates, again, that the level of research from Africa is very low, but also that there is a lack of applied research from the local knowledge base. The co-citation analyses show that the African-related research focuses on the economic burden of crashes, crash issue identification, injuries and, to some extent, injury prevention. Cultural differences are also researched. International works focus on the impact of interventions as well as broader-level data analyses. By implication, the African research is still focused on identification of the key issues in driver behaviour, whereas it appears that international research has gone beyond this into intervention identification, implementation and analysis of effectiveness. The co-occurrence of keywords indicates that, whilst themes are similar in the African and international research, the linkages between African-related research themes tend to be weak, suggesting that the themes lack broad-based applicability, making it difficult to connect and form a more cohesive body of work. The thematic analysis reveals that themes such as driver behaviour are universal across both research areas, but that issues related to networks and systems approaches are missing in the African-related literature, as is model design, again suggesting the need for broader-based research and solution-driven approaches. Analysis of the highest cited papers reinforces the need for broader-based research. Lagarde [48], for example, considered the economic burden of traffic accidents to Africa as a whole; Kouabenan [49] provided a wide perspective on all the driver causation factors in road crashes; Nordfjærn, Jørgensen and Rundmo [40] considered cross cultural differences; Chokotho, Matzopoulos and Myers [53] considered the quality of traffic injury data; and Verster and Fourie [56] provided a high level overview of factors contributing to traffic accidents. This suggests a need for research which is applicable across a range of environments.

## 5. Conclusions

This research was limited to two bibliometric analyses and document reviews from the Web of Science database, which were focused on driver behaviour and traffic safety in Africa and globally. The results reveal several research gaps in the African-related studies, which then assist in identifying a future research agenda.

- Many countries have severe driver-related traffic safety issues and high fatality rates, yet little to no research is emanating from these countries. Whilst high-level works on Africa reside in organisations such the World Health Organisation and United Nations, little exists to provide country overviews. Research is needed to provide a broad perspective of driver-related road safety issues on a country-by-country basis, particularly as the evidence suggests that different issues are present in different regions and cultures.
- Cross-country comparisons would therefore also be of value, so that interventions can be identified that suit specific environments.
- The requirement for road crash reduction is clearly outlined in the SDGs, yet little research has been conducted in terms of linking the issue of driver behaviour to sustainable development. This type of research would inform policymakers of the clear linkage between accident reduction and better socio-economic conditions, and show, therefore, the need to implement driver behaviour interventions.
- Research is required that goes beyond issue identification and into potential interventions and solutions. Research into the efficacy of driver modification interventions is an appropriate evolution.
- Data on driver behaviour and road safety are sparse in Africa. Consideration needs to be given to the development of continent-wide and country-specific databases. There is an accompanying need for statistical analysis and modelling.
- No studies have considered the issue of driver behaviour and traffic safety on the continent from a policy perspective. Although several countries have adopted policies such as the safe systems approach, little is known about the policy approaches in various countries, policy implementation or effectiveness of policies. Policy-related research could also consider policy requirements for specific environments.

These research themes are aimed at addressing the research gaps in driver behaviour and road safety in Africa, specifically the need for far greater levels of research and data, particularly for countries with little to no current research in this field. Country-specific issues need to be identified so that comprehensive interventions can be determined, implemented and evaluated. Inclusive research in this area is likely to broaden the knowledge base, especially the local knowledge base, to enable more effective interventions and policy directions.

## Figures and Tables

**Table 1:** Main information about the data.

Description	Results
Timespan	1996:2022
Sources (Journals, Books, etc.)	65
Documents	96
Annual Growth Rate %	5.48
Document Average Age	6.78
Average citations per doc	16.22
References	3682
DOCUMENT TYPES	
article	82
article; early access	1
article; proceedings paper	2
review	11
DOCUMENT CONTENTS	
Keywords Plus (ID)	300
Author’s Keywords (DE)	334
AUTHORS	
Authors	282
Authors of single-authored docs	14
AUTHORS COLLABORATION	
Single-authored docs	14
Co-Authors per Doc	3.3
International co-authorships %	42.71

Source: Author’s own, based on Web of Science search.

**Table 2:** Citations and publications per journal.

Element	h_Index	g_Index	m_Index	TC	NP	PY_Start
Accident Analysis and Prevention	7	9	0.292	377	9	1999
Traffic Injury Prevention	6	6	0.6	102	6	2013
IATSS Research	3	3	0.375	25	3	2015
Journal of Transport and Health	3	3	0.429	27	3	2016
Transportation Research Part F-Traffic Psychology and Behaviour	3	3	0.3	24	3	2013
International Journal of Injury Control and Safety Promotion	2	3	0.154	40	3	2010
Journal of Transport Geography	2	2	0.167	21	2	2011
Pan African Medical Journal	2	2	0.222	11	2	2014
PLOS One	2	2	0.167	41	2	2011
Safety Science	2	3	0.143	140	3	2009
SAMJ South African Medical Journal	2	2	0.182	6	3	2012
South African Journal of Science	2	2	0.095	21	2	2002

Source: Author’s own, based on Web of Science search. (TC = total citations, NP = number of publications, PC_start = start year).

**Table 3:** Most influential authors.

Element	h_Index	g_Index	m_Index	TC	NP	PY_Start
Rundmo T	4	4	0.286	262	4	2009
Renner W	3	3	0.15	196	3	2003
Nordfjaern T	3	3	0.25	141	3	2011
Hyder AA	3	3	0.3	128	3	2013
Lagarde E	2	2	0.125	123	2	2007
Lund IO	1	1	0.071	121	1	2009
Bachani AM	3	3	0.3	110	3	2013
Kouabenan DR	1	1	0.04	106	1	1998
Peltzer K	2	2	0.1	104	2	2003
Jorgensen S	2	2	0.167	99	2	2011
Boikhutso N	1	1	0.111	73	1	2014
Hofman KJ	1	1	0.111	73	1	2014
Wesson HKH	1	1	0.111	73	1	2014
Bachoo S	1	1	0.1	71	1	2013
Bhagwanjee A	1	1	0.1	71	1	2013
Govender K	1	1	0.1	71	1	2013
Nordberg E	1	1	0.043	71	1	2000
Forjuoh SN	1	1	0.042	70	1	1999
Mock CN	1	1	0.042	70	1	1999
Rivara FP	1	1	0.042	70	1	1999
Factor R	1	1	0.067	63	1	2008
Mahalel D	1	1	0.067	63	1	2008
Yair G	1	1	0.067	63	1	2008
Charles AG	2	2	0.182	50	2	2012
Simsekoglu O	1	1	0.111	42	1	2014

Source: Author’s own, based on Web of Science search.

**Table 4:** Author affiliations.

Affiliation	Country	Articles
Univ Alabama	United States	20
Univ Pretoria	South Africa	15
Univ Cape Town	South Africa	10
Univ Witwatersrand	South Africa	7
Kwame Nkrumah Univ Sci and Technol	Ghana	6
Norwegian Univ Sci and Technol	Norway	6
Univ N Carolina	United States	6
Univ South Africa	South Africa	6
Johns Hopkins Bloomberg Sch Publ Hlth	United States	5
Lagos State Univ	Nigeria	5
Univ Kwazulu Natal	South Africa	5
Beit Cure Hosp	Zambia	4
Columbia Univ	United States	4
Kamuzu Cent Hosp	Malawi	4
Kumasi Tech Univ	Ghana	4
Univ Abomey Calavi	Benin	4
Univ Nairobi	Kenya	4
Univ Western Ontario	United States	4
Fed Med Ctr	Nigeria	3
France	France	3
Inst Rd Safety Res Swov	Netherlands	3
Northwest Univ	South Africa	3
Stellenbosch Univ	South Africa	3
Univ E Anglia	United Kingdom	3
Univ Free State	South Africa	3
Univ Leeds	United Kingdom	3
Univ Saskatchewan	Canada	3
Univ Stellenbosch	South Africa	3
Univ Washington	United States	3

Source: Author’s own, based on Web of Science search.

**Table 5:** Most cited countries.

Country	Total Citations	Average Article Citations
South Africa	299	12.46
USA	270	14.21
France	229	76.33
Norway	227	56.75
Ghana	113	22.60
Kenya	93	31.00
Israel	63	63.00
Malawi	55	18.33
United Kingdom	54	5.40
Turkey	42	42.00
Switzerland	39	19.50
Nigeria	32	4.57
Italy	12	6.00
Canada	8	4.00
Botswana	5	5.00
China	5	5.00
Netherlands	5	5.00
Cameroon	3	3.00
Benin	1	0.50
Germany	1	1.00
Rwanda	1	1.00
India	0	0.00
Russia	0	0.00

Source: Author’s own, based on Web of Science search.

**Table 6:** Top 10 cited papers.

Rank	Authors	Local Citations	Global Citations	LC/GC Ratio	Summary of Paper
1	Lagarde [48]	8	101	7.92	The paper considers the high level of injury and costs associated with road accidents in Africa and determines that a lot more research is required into the phenomenon.
2	Kouabenan [49]	6	106	5.66	The author asserts that accident causes may be linked to characteristics inherent to social groups: beliefs, value systems, norms, experiences in common, attitudes, roles, social and technical practices, etc. Culturally determined bias seems to affect the perception of risk and the causes of accidents. Considering causal attributes in the Ivory Coast (West Africa), the research shows that fatalistic beliefs and mystical practices influence the perception of accidents and consequently incite one to take more risks and neglect safety measures.
3	Jones et al. [50]	4	15	26.67	The paper considered professional opinion from Ethiopia, Kenya and Ghana and found evidence of significant negative public health outcomes associated with crashes and pollution attributable to poor mechanical conditions of rural transport vehicles. Risky driving behaviours were reported to be an important source of injury from rural road crashes.
4	du Plessis, Hlaise and Blumenthal [51]	2	7	28.57	This study examined blood alcohol concentrations in different road users in Ga-Rankuwa, South Africa, and found that the majority of victims were adult males. Female victims also showed high blood alcohol levels. The results were relevant for drivers and pedestrians.
5	Maldonado, Mitchell, Taylor and Driver [52]	1	9	11.11	The study considered the sleep, work schedules and accident risk amongst long-haul truck drivers and established that sleep deprivation and illegally long working hours led to driving behaviour that could result in accidents.
6	Nordfjærn, Jørgensen and Rundmo [40]	1	81	1.23	The study considers cross-cultural differences in road traffic risk perception, risk sensitivity and risk willingness in Norway, Russia, India, Ghana, Tanzania and Uganda. Sub-Saharan Africa showed higher road traffic risk perceptions and sensitivity than respondents from Norway, Russia and India. Participants from sub-Saharan Africa and India show safer attitudes to speaking out to an unsafe driver, rule violations and sanctions, attitudes towards pedestrians, and traffic rules and knowledge.
7	Chokotho, Matzopoulos and Myers [53]	1	18	5.56	The study assessed whether the quality of the available road traffic injury (RTI) data was sufficient for determining the burden of RTIs in the Western Cape Province and for implementing and monitoring road safety intervention. The study found extensive data quality problems, implying that any findings had to be interpreted with considerable caution.
8	Matheka, Alkizim, Kipsaina and Witte [54]	1	7	14.29	Motorcycle injuries contribute to a substantial number of deaths and hospital admissions in Kenya. The majority of motorcycle-related injuries in Thika town occur among young, productive, working-age male drivers. A high proportion of injuries are due to negligence in riding while not wearing protective equipment, compounded by lack of injury prevention education.
9	Solagberu et al. [55]	1	11	9.09	The study considered pedestrian injuries in Lagos, Nigeria, and established patterns in injuries and causes. Whilst not exclusively focused on driver behaviour, the results suggested that poor driver adherence to safety precautions and signage was one of the major causes for pedestrian injuries.
10	Verster and Fourie [56]	1	12	8.33	Accident information was reviewed by looking at three general contributing factors: human factors, road and environmental factors and the vehicles involved in the accident. Human factors contributed to almost 80% of the fatal accidents on South African roads, for which 52.5% of these incidents related to jaywalking and 11.6% to speeding.

Source: Author’s own, based on Web of Science search.

## Data Availability

Data available from Web of Science using the search terms (traffic OR road safety) AND (driv*) AND Africa.
